# Conformational Dynamics of the Soluble and Membrane-Bound Forms of Interleukin-1 Receptor Type-1: Insights into Linker Flexibility and Domain Orientation

**DOI:** 10.3390/ijms23052599

**Published:** 2022-02-26

**Authors:** João P. Luís, Ana I. Mata, Carlos J. V. Simões, Rui M. M. Brito

**Affiliations:** 1 Coimbra Chemistry Center—Institute of Molecular Sciences (CQC-IMS), Department of Chemistry, University of Coimbra, 3004-535 Coimbra, Portugal; ana.mata@student.uc.pt (A.I.M.); csimoes@qui.uc.pt (C.J.V.S.); 2 BSIM Therapeutics, Instituto Pedro Nunes, 3030-199 Coimbra, Portugal

**Keywords:** interleukin-1, interleukin-1 receptor type 1, flexible linker, molecular dynamics

## Abstract

Interleukin-1 receptor type 1 (IL-1R1) is a key player in inflammation and immune responses. This receptor regulates IL-1 activity in two forms: as a membrane-bound form and as a soluble ectodomain. The details and differences between the conformational dynamics of the membrane-bound and the soluble IL-1R1 ectodomains (ECDs) remain largely elusive. Here, we study and compare the structural dynamics of the soluble and membrane-bound IL-1R1-ECDs using molecular dynamics (MD) simulations, focusing on the flexible interdomain linker of the ECD, as well as the spatial rearrangements between the Ig-like domains of the ECD. To explore the membrane-bound conformations, a full-length IL-1R1 structural model was developed and subjected to classical equilibrium MD. Comparative analysis of multiple MD trajectories of the soluble and the membrane-bound IL-1R1-ECDs reveals that (i) as somewhat expected, the extent of the visited “open-to-closed” transitional states differs significantly between the soluble and membrane-bound forms; (ii) the soluble form presents open-closed transitions, sampling a wider rotational motion between the Ig-like domains of the ECD, visiting closed and “twisted” conformations in higher extent, whereas the membrane-bound form is characterized by more conformationally restricted states; (iii) interestingly, the backbone dihedral angles of residues Glu202, Glu203 and Asn204, located in the flexible linker, display the highest variations during the transition between discrete conformational states detected in IL-1R1, thus appearing to work as the “central wheel of a clock’s movement”. The simulations and analyses presented in this contribution offer a deeper insight into the structure and dynamics of IL-1R1, which may be explored in a drug discovery setting.

## 1. Introduction

The interleukin-receptor type 1 (IL-1R1) belongs to a superfamily of receptors with pivotal roles in the immune system, positioned at the “epicenter” of the inflammatory signaling networks [1,2]. IL-1R1 is an 80 kDa transmembrane protein featuring an extracellular domain (ECD) containing three Ig-like domains (D1, D2 and D3), responsible for ligand recognition, a transmembrane (TM) α-helix, and a cytoplasmic TIR domain responsible for the initiation of intracellular signaling [3,4,5,6]. This receptor also exists as a soluble ectodomain circulating form, following proteolytic release of the ECD of membrane-anchored IL-1R1 via matrix metalloproteases. Both forms of IL-1R1 are biologically active, regulating the inflammatory response through agonistic and antagonistic modulation of cytokine activity. For this purpose, the ectodomain (henceforth referred to as IL-1R1-ECD) of soluble and membrane-bound forms is vital for ligand recognition and binding [7,8,9]. Therefore, the understanding of the structural intricacies of both IL-1R1-ECD forms is pivotal not only to pinpoint the general mechanisms of signal activation and inhibition via the IL-1 cytokines, but also to detect or predict putative druggable regions for pharmacological intervention. The “puzzling nature” of soluble and membrane-bound IL-1R1-ECDs rises up questions of whether there are differences between the conformational landscapes visited by the two forms and whether such differences may uphold the assumption that the two states may hold distinct functions.

Vigers et al. reported the first crystallographic structure of the IL-1R1-ECD bound to IL-1β, solved at 2.5 Å resolution (Protein Data Bank [PDB] entry 1ITB) [10]. Such data provided a structural picture of IL-1R1-ECD’s organization: (i) domains 1 (D1) and 2 (D2) are tightly joined with a disulfide bridge that confers stability and structural rigidity to these domains, and (ii) domain 3 (D3) is connected via a flexible linker to D2, which is able to move freely with respect to D1 and D2 [11]. The flexibility of this linker is crucial for positioning D3 for cytokine binding, allowing the formation of stable complex and then the recruiting of the IL-1 receptor accessory protein (IL-1RAcP) [8,12]. Interestingly, a structural complex of the IL-1R1-ECD with a small antagonist peptide is available (PDB entry 1G0Y), showing a 170° rotation of D3 relative to a reference D3 in the IL-1R1:IL-1β complex (PDB entry 1ITB), and thus exposing an unexpected binding mode for the peptide [11]. Recently, structural analyses employing a combination of small-angle X-ray scattering (SAXS) measurements with molecular dynamics (MD) simulations of the IL-1R1-ECD revealed an ensemble of closed conformations where D3 was also rotated, highlighting the role of this linker’s flexibility-dependent function on two structurally similar IL-1R1 ectodomains—IL-1RAcP-ECD and IL-18Rβ-ECD [13]. Such structural insights make us raise new questions and incite us to investigate the spectrum of linker conformations explored by the soluble IL-1R1-ECD in comparison with the membrane-bound IL-1R1-ECD: (i) are conformational dynamics of the linker majorly affected by the membrane environment? (ii) do IL-1R1 structural and conformational preferences differ between soluble and membrane-bound IL-1R1-ECDs?

While extensive structural works on the soluble ECD portions of IL-1R1 have provided critical insights about the specificities of ligand binding to this protein, lack of a full-length receptor structure has hindered a comprehensive overview of the overall architecture and IL-1R1 domain arrangements. In this regard, in this study we attempt to predict the structural organization of full-length IL-1R1. Starting off from a high-quality crystal structure of the apo form of IL-1R1-ECD, we compare the conformational ensembles sampled by MD simulations of the full-length (IL-1R1 ECD-TM-TIR) membrane model with those sampled by the soluble protein (IL-1R1-ECD). To the best of our knowledge, this is the first study of IL-1R1 dynamics in both the soluble and the membrane-bound forms, and based on ten individual 600 ns-long MD runs totaling 6 μs of simulation.

Altogether, our simulations expand the current understanding of the dynamic features of the soluble and membrane-bound forms of IL-1R1, corroborating and mounting on the observations of other studies [13,14,15]. The incorporation of dynamic features, such as the ones highlighted in the present work, provide a better understanding of the differences between the soluble and membrane-bound IL-1R1-ECD conformational landscape.

## 2. Results

**Figure 1:**
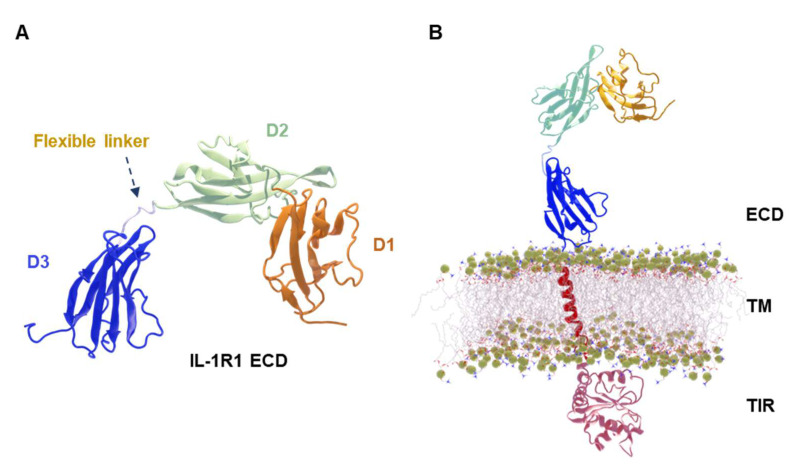
Initial configurations of soluble and full-length membrane-bound interleukin-1 receptor type 1 (IL-1R1). (**A**) Structural organization of the soluble IL-1R1. Ig-like domains are labeled as D1, D2 and D3, and the arrow indicates the 6 amino acid flexible linker. (**B**) Overall architecture of the full-length IL-1R1 in a POPC membrane.

**Figure 2:**
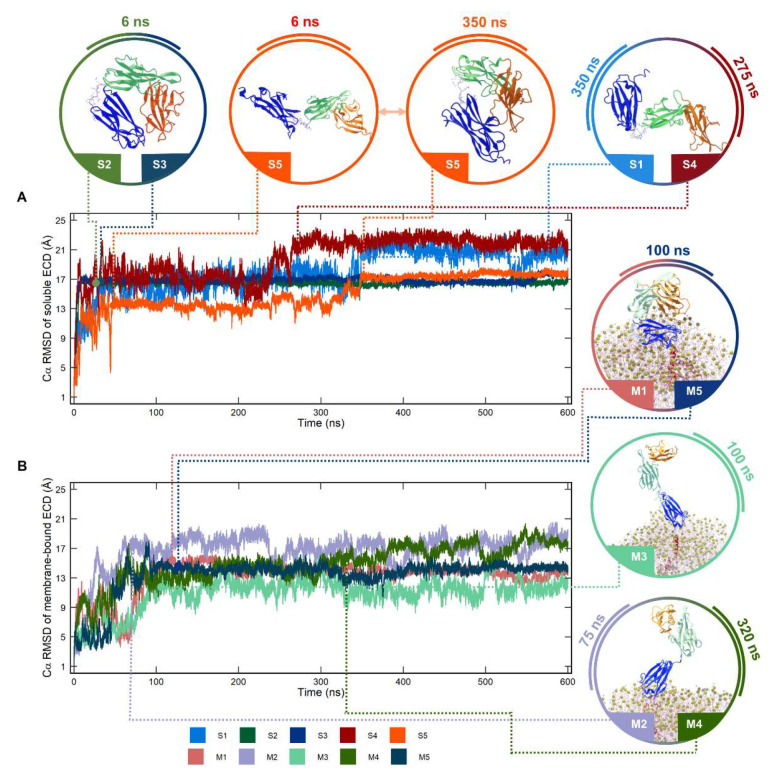
Variation of the root mean square deviation (RMSD) of all Cα-atoms of the extracellular domain of interleukin-1 receptor type 1 (IL-1R1-ECD). (**A**) Five 600-ns soluble IL-1R1-ECD MD simulations (S1, S2, S3, S4 and S5); (**B**) five 600-ns membrane-bound IL-1R1-ECD MD simulations (M1, M2, M3, M4 and M5).

As a means to characterize the conformational behavior of the membrane-bound IL-1R1-ECD, in this study we developed a structural model in which the full-length IL-1R1 is anchored to a 1-palmitoyl-2-oleoyl-sn-glycero-3-phosphocholine (POPC) membrane. For both the soluble and membrane-bound IL-1R1-ECDs, five independent 600 ns-long MD simulations, at 303 K, were performed, totaling 3 μs for each system. Figure 1 depicts the overall structures of both the soluble, X-ray derived IL-1R1 (Figure 1A) and the modeled, membrane-bound, full-length IL-1R1 (Figure 1B). The structures were validated through PROCHECK [16] and ProSA-web server [17] (see Supplementary Table S1, Figure 1 and Figure 2), and secondary structure assignments for the IL-1R1-ECD X-ray structure (PDB entry 4GAF) and across the MD trajectories were computed with the DSSP algorithm [18] (see Supplementary Figures S3–S5). The structural stability of the POPC membrane in the presence of full-length IL-1R1 was examined by computing the surface area occupied by each lipid (area per lipid), lipid order parameters and mass density profiles across the membrane (see Supplementary Figure S6), demonstrating, overall, good agreement with structural parameters for a pure POPC bilayer—as determined experimentally and from MD simulations [19,20,21,22,23]. A detailed analysis of the simulations is presented next.

### 2.1. Conformational Dynamics of Soluble and Membrane-Bound IL-1R1-ECDs

The root mean square deviation (RMSD) and the root mean square fluctuation (RMSF) were computed for the soluble and membrane-bound IL-1R1-ECDs after alignment of the coordinates of Cα-atoms from all systems, using the open IL-1R1-ECD X-ray structure (PDB entry 4GAF) as reference. Analysis of the RMSD profiles (Figure 2) reveals significant conformational changes compared to the starting structure for the soluble IL-1R1. In the 2nd and 3rd replicas (S2 and S3), the ECD adopts a stable, closed conformation early in the MD trajectory, i.e., after ≈ 6 ns (average Cα-RMSD of 16.5 ± 0.02 Å and 16.9 ± 0.04 Å, respectively), mediated by D1/D3 β-sheet interactions, preserving this rigid state throughout the rest of the simulated time. Two main conformational changes in IL-1R1-ECD are observed in S5: (i) during the first 30 ns of simulation, it adopts an open, extended configuration, and (ii) it then undergoes an open-to-closed transition with significant structural rearrangements on D3 between 30 and 350 ns—with the respective RMSD values afterwards approaching those observed in the two replicas sampling the stable, closed state. The remaining two runs, S1 and S4, show the ECD exhibiting wider variations in terms of RMSD profiles, mostly characterized by rapid increasing and fluctuating values until the end of the simulation. The mean Cα-RMSD of S1 and S4 is 18.8 ± 0.22 Å and 20.6 ± 0.24 Å, respectively, denoting a larger departure from the initial structure on average, compared with the other replicas. Interestingly, in S1 and S4 the D3 region undergoes a rotational motion relative to the other two domains, allowed by the flexible linker connecting D2 and D3, with the ectodomain adopting a “twisted” conformation that looks considerably distinct from both the closed and the initially open conformations.

The RMSD profiles of the membrane-bound IL-1R1-ECD essentially follow a similar trend to that of soluble ECD. Interestingly, during the first 100 ns of the M1 and M5 trajectories, IL-1R1-ECD explores an open configuration more extensively before transitioning into a more stable closed state. This is in contrast with what is observed in replicas of the soluble system transitioning into a similar closed state after around 6 ns of simulation (S2 and S3). The mean Cα-RMSD values in M1 and M5 are of 14.1 ± 0.07 Å and 14.1 ± 0.06 Å, respectively, suggesting that the membrane-bound ECD shows lower deviations than the soluble ECD. Higher average RMSD values of 16.7 ± 0.3 Å and 14.8 ± 0.3 Å were computed for trajectories M2 and M4, where IL-1R1-ECD adopts an extended and open-twisted conformation in the later snapshots of the simulations. However, in the M3 trajectory, the ectodomain remains in a remarkably stable open conformation throughout the whole simulation length, without the occurrence of structural rearrangements around the flexible linker—resulting in a mean Cα-RMSD of 10.9 ± 0.2 Å.

The MD trajectories demonstrate that the conformational stability of IL-1R1-ECD is, on average, higher when bound to a lipid-bilayer, in comparison to the soluble IL-1R1-ECD. Other structural properties, such as the radius of gyration (Rg), intramolecular hydrogen bonds (HB_intra_) and solvent accessible surface area (SASA) were evaluated. Table 1 reports results demonstrating that the ectodomains adopting a closed conformation in S2, S3, M1 and M5 present similar Rg values, whereas a clear increment in Rg is observed for the systems exploring open conformations. Interestingly, in the S5 trajectory, the transition from the open to a somewhat distinct closed configuration is reflected in a slightly higher Rg when compared to the Rg calculated across those trajectories, where the ECD largely adopted closed conformations. In S5, less intramolecular hydrogen bond formation is verified in comparison with those trajectories sampling closed conformations, suggesting that the hydrogen bond framework of IL-1R1-ECD may be altered due to the rotational motions of the linker across the earlier 350 ns. The higher number of HB_intra_ observed for the IL-1R1-ECD closed states points out that this conformation could be thermodynamically more stable. The closed conformations also expose higher SASA across the soluble and the membrane-bound IL-1R1-ECDs (SASA values between 172.7 and 176.4 nm^2^), which is consistently below the SASA of the open conformations (SASA values between 178.7 and 186.3 nm^2^). These structural properties extracted for the simulations are in good agreement with the ones computed for the closed (PDB entry 1G0Y, SASA of 174.4 nm^2^) and open (PDB entry 4GAF, SASA of 179.4 nm^2^) IL-1R1-ECD X-ray structures. Intriguingly, the number of HB_intra_ observed for the closed conformations was systematically lower than the HB_intra_ value of the IL-1R1-ECD closed crystal structure, indicating the transitional pathway to this structure as not having been sampled during the simulation timescale.

**Figure 3:**
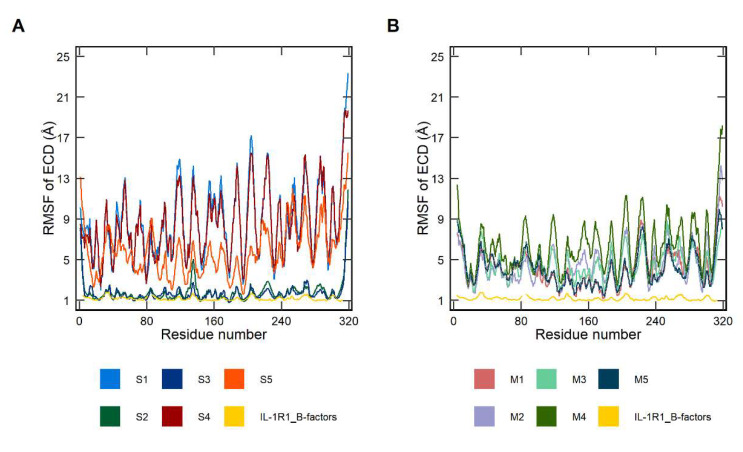
Variation of the root mean square fluctuation (RMSF) of all Cα-atoms profiles as a function of residue number for the extracellular domain of interleukin-1 receptor type 1 (IL-1R1-ECD). (**A**) Five 600-ns soluble IL-1R1-ECD MD simulations (S1, S2, S3, S4 and S5); (**B**) five 600-ns membrane-bound IL-1R1-ECD MD simulations (M1, M2, M3, M4 and M5). The line colored in yellow represents the fluctuations derived from the crystallographic B-factors of IL-1R1-ECD (PDB entry 4GAF).

Figure 3 shows the RMSF of Cα-atoms compared with RMSF values derived from the crystallographic B-factors of IL-1R1 (PDB entry 4GAF). The latter were obtained using the relationship RMSF = (3B/8π^2^)^1/2^. The RMSF values correspond mainly to the N- and C-terminal tails of the ECD and to loop regions. It is clear that both soluble and membrane-bound ECDs present significant mobility differences between replicas of the two systems. The MD trajectories where the soluble IL-1R1-ECD undergoes a conformational transition into a closed state (S2 and S3) exhibit modest and nearly superimposable Cα-atom fluctuations, whereas larger fluctuations are detected in the MD trajectories where the membrane-bound IL-1R1-ECD majorly explore a closed configuration (M1 and M5). On the contrary, in those trajectories where IL-1R1-ECD adopts open-twisted configurations, significantly larger fluctuations on the Cα-atoms of all residues are observed, with a somewhat expected sharp peak observed between residues 201 and 206. These residues comprise the flexible linker between D2 and D3. The higher RMSF values indicate that all three domains are prominently changing their spatial orientations throughout the simulations, due to the interdomain movements mediated by the linker. Comparisons between the soluble and the bilayer-bound ECD indicate that the former exhibits higher structural flexibility and more pronounced configurational transitions than the latter. This is likely due to the higher conformational freedom enjoyed by the soluble ECD, allowing it to explore a diversity of backbone conformations in higher extent compared to the membrane-bound ECD.

**Figure 4:**
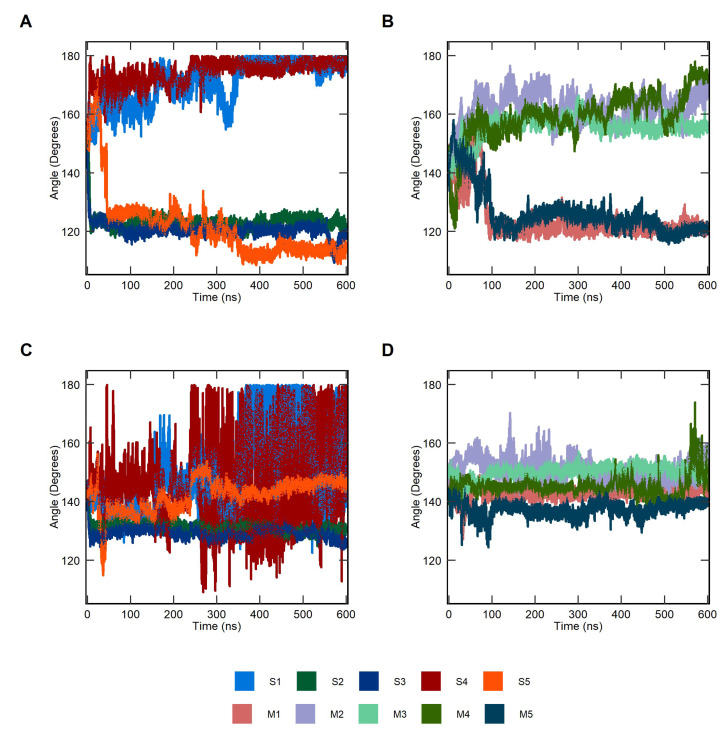
Distributions of the interdomain hinge angles of the extracellular domain of interleukin-1 receptor type 1 (IL-1R1-ECD) throughout the whole simulation length. (**A**,**C**) D1-D3 and D2-D3 hinge angles, respectively, for the soluble IL-1R1-ECD (S1, S2, S3, S4 and S5) and (**B**,**D**) D1-D3 and D2-D3 hinge angles, respectively, for the membrane-bound (M1, M2, M3, M4 and M5) IL-1R1-ECD.

To get further insight into the structural dynamics of both IL-1R1-ECD forms, the interdomain hinge angles between D1-D3 and D2-D3 were analyzed. The flexible linker mediates conformational changes in solution, with hinge-bending and hinge-twisting motions moving the domains together (closed conformation) and apart (open conformation). The hinge angle was computed by measuring the relative displacement between the center of the masses of the two domains in the XY-plane. Figure 4 illustrates the comparison of the interdomain hinge angle (ϕ) distributions for the soluble and the membrane-bound IL-1R1-ECDs within the simulation timescale. For both forms, two defined clusters of D1-D3 hinge angles are accessible, sampling both the closed and open conformations. In the soluble structure, the majority of D1–D3 angles for closed conformations (prevalent in S2, S3 and S5) is concentrated within the 110° to 125° range, while the range on the open-twisted conformations (sampled by S1 and S4) is broadened, ranging between 150° to 180°. In line with the RMSD and Rg results, the S5 trajectory samples open extended conformations during the first 30 ns, reaching its largest angle of 171.5° at 23.4 ns, which gradually closes up to ≈115°. Likewise, comparable patterns are observed for the membrane-bound ECDs. However, two important differences stand out when comparing to the soluble state: (i) attachment to the membrane appears to limit the sampling of open-to-closed transitions, solely converging to a similar hinge angle (ϕ ≈120°) after ≈100 ns (M1 and M5), and (ii) a smaller hinge angle distribution (fluctuations between 150° and 172°) is observed in the open conformations (mostly sampled by M2, M3 and M4) when compared to the corresponding structures of the soluble ECD (mostly sampled by S1 and S4). This observation is in agreement with the computed hinge angles distributions of D2-D3, where the soluble ECD exhibits larger interdomain movements in S1 and S4, whereas M2, M3 and M4 sample a narrow angle distribution.

### 2.2. Principal Component Analysis of Soluble and Membrane-Bound IL-1R1-ECDs MD Trajectories

**Figure 5:**
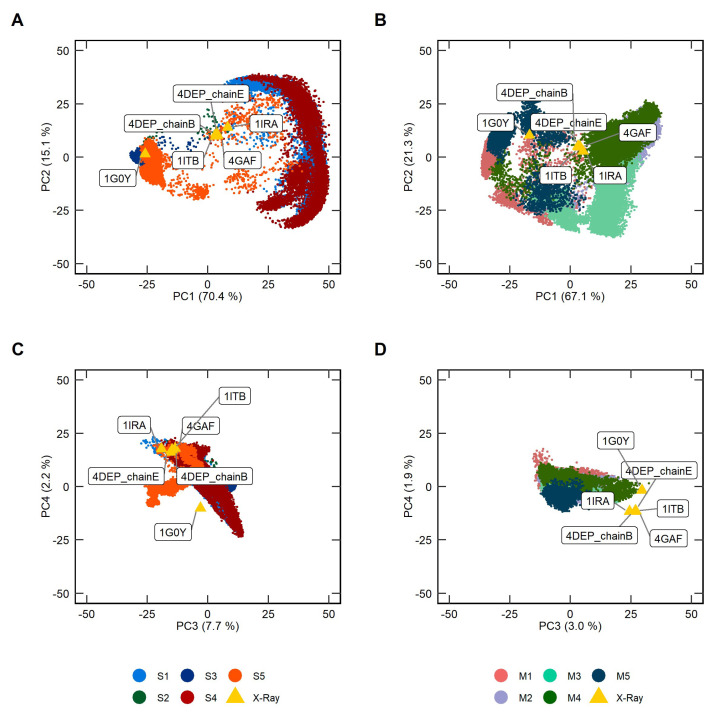
Projections of the conformations adopted by the extracellular domain of interleukin-1 receptor type 1 (IL-1R1-ECD) onto the PC1-4 essential subspace. The experimental structures of IL-1R1-ECD (shown in yellow triangles) used as reference points are depicted with text labels. Sampled areas of (**A**,**C**) the five soluble (S1, S2, S3, S4 and S5) space; (**B**,**D**) five membrane-bound (M1, M2, M3, M4 and M5) IL-1R1-ECD MD simulations, in the two-dimensional PC1-PC2 and PC3-PC4 space.

Principal component analysis (PCA) was performed on protein backbone atoms to help focus on the differences of conformational dynamics between the soluble and membrane-bound IL-1R1-ECDs. In essence, PCA converts a set of correlated observations, e.g., movements of selected atoms in a system, to a set of principal components (PCs) that are linearly independent, containing, for example, the dominant trends explaining rigid-body domain motions. The 2D plots of Figure 5 depict the distribution of conformations along the PC1/PC2 and PC3/PC4 for both IL-1R1-ECD forms extracted from the ten independent MD trajectories. The positions for the five available experimental structures of the IL-1R1-ECD were reported to map reference points onto the subspace spanned by the essential PCs. The resulting PCs analysis scree plot, indicating the proportion of variance accounted by the PCs, is provided in Supplementary Figure S7. The porcupine plots of the four most representative PCs are presented in Supplementary Figure S8. In both IL-1R1-ECD forms, the first two eigenvectors (PC1 and PC2) obtained from the PC analysis capture more than 85% of the total variance, suggesting that these vectors approximately describe the essential subspace of both systems. Movement along PC1 describes a collective motion from the closed to the open state, resulting in a change in the hinge angles, whereas the PC2 pertain the collective twisting motions of domains D1-D2 with respect to D3. PC3 and PC4 each account for only 2.2–7.7% and 1.9–3.0% of the observed variance in the soluble and membrane-bound ECD structures, respectively. The PCs represent motions of the D3 domain twisting about the flexible linker.

As observed from Figure 5, the conformational landscape explored by the two IL-1R1-ECDs reveals differences: (i) most of the soluble ECD conformations were mapped into two distinct regions of the conformational space, with a small number of intermediate conformations connecting them, i.e., adopting closed conformations where D1 and D3 are tightly packed against each other (−25 < PC1 < 15) and open-twisted states (20 < PC1 < 50); (ii) in the membrane-bound IL-1R1-ECD a higher number of intermediate open states between the closed and open-twisted conformations is observed. Furthermore, the area spanned by PC1 and PC2 was much larger in the case of the soluble IL-1R1-ECD in comparison to the membrane-bound form, suggesting larger amplitude of motion and, thus, flexibility of the soluble ECD. As mentioned above, the RMSD and RMSF curves, as well as the hinge angle distribution for the membrane-bound IL-1R1-ECD also appear to support this interpretation (Figure 2, Figure 3 and Figure 4). Interestingly, it can be observed that both IL-1R1-ECD forms sampled (in the first two PCs) the conformational space encompassing the X-ray structures deposited in the PDB. However, non-overlapping areas of the conformational space explored by the MD simulations and the configurations portrayed by the X-ray structures were observed in PC3 and PC4, despite their lower contribution. This is clearer for the membrane-bound IL-1R1-ECD, which explored different regions in PC3 and PC4 when compared to the structures determined experimentally, whilst the soluble form was not able to sample the closing of the ECD with a 170° rotation of D3 relative to D1-D2, as verified in the closed antagonist-bound IL-1R1-ECD (PDB entry 1G0Y). These results point toward notable differences in the twisting motions of the two IL-1R1-ECD forms.

### 2.3. Clustering Analysis of Soluble and Membrane-Bound IL-1R1-ECDs Conformations

**Figure 6:**
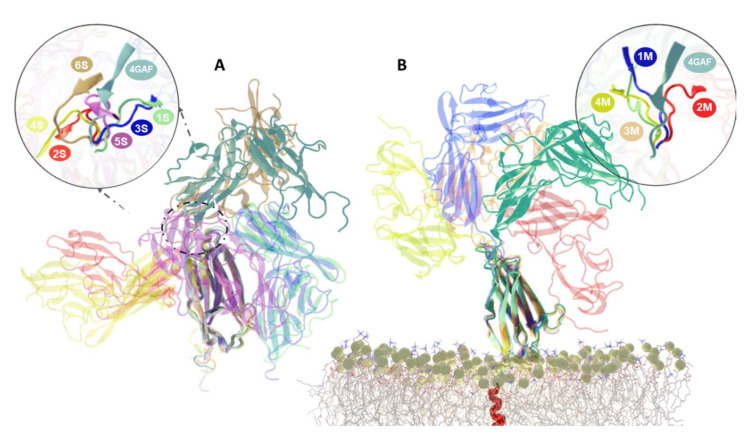
Cluster representative structures of the extracellular domain of interleukin-1 receptor type 1 (IL-1R1-ECD) with a zoomed view of flexible linker orientations, derived from a clustering approach for linker conformations based on backbone dihedral angles (φ, ψ). Representative conformations for (**A**) the soluble and (**B**) membrane-bound IL-1R1-ECD MD simulations. All structures were aligned to the D3 domain of the X-ray structure of open IL-1R1-ECD configuration (PDB entry 4GAF), which is shown in dark green. Represented clusters of soluble ECD: Cluster 1 [1S] (green); Cluster 2 [2S] (red); Cluster 3 [3S] (blue); Cluster 4 [4S] (yellow); Cluster 5 [5S] (purple); Cluster 6 [6S] (brown). Represented clusters of membrane-bound ECD: Cluster 1 [1M] (blue); Cluster 2 [2M] (red); Cluster 3 [3M] (orange); Cluster 4 [4M] (yellow).

**Figure 7:**
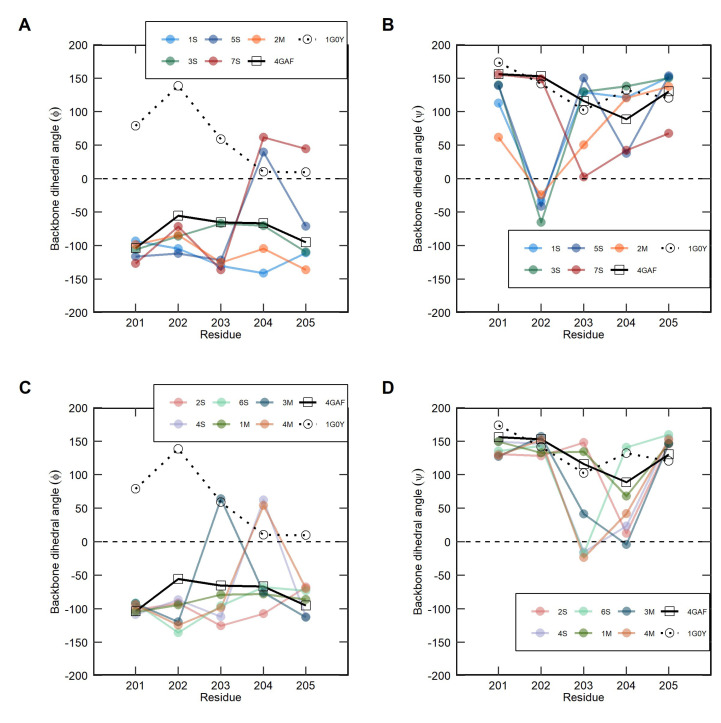
Comparison of the calculated linker backbone dihedral angles, ϕ (**A**,**C**) and ψ (**B**,**D**), for the representative clusters of the extracellular domain of interleukin-1 receptor type 1 (IL-1R1-ECD) with those measured in the X-ray structures. The black solid line denotes the open IL-1R1-ECD (PDB entry 4GAF) and the dashed line denotes the closed, twisted IL-1R1-ECD conformation (PDB entry 1G0Y). Represented clusters of soluble ECD: Cluster 1 [1S] (light blue); Cluster 2 [2S] (red); Cluster 3 [3S] (dark green); Cluster 4 [4S] (mauve); Cluster 5 [5S] (blue); Cluster 6 [6S] (light green). Represented clusters of membrane-bound ECD: Cluster 1 [1M] (green); Cluster 2 [2M] (orange); Cluster 3 [3M] (blue); Cluster 4 [4M] (brown).

Clustering analysis focused on backbone dihedral angles (φ, ψ) of selected linker residues has been performed in order to identify and compare discrete ECD configurations selected by the soluble and the membrane-bound IL-1R1 throughout the simulations. Figure 6 and Figure 7 provide information on the cluster populations and backbone dihedral angles (φ, ψ) of residues Leu201, Glu202, Glu203, Asn204 and Lys205 for each cluster representative. It is worth emphasizing that these dihedral angles presented in Figure 7 remain within the most favored or additional allowed regions of the Ramachandran plot (see Supplementary Figure S9). For the sake of illustration, only clusters encompassing 5% or more of the total population of structures are represented in Figure 6. The representative IL-1R1-ECD conformations derived from the respective linker backbone torsion angles were aligned to the D3 of the crystal structure (PDB entry 4GAF) for visualization and comparison purposes. The sin- and cos-transformed scaled dihedral angles were classified into clusters using the partition around medoids (PAM) algorithm. The average silhouette method was used to estimate the optimal number of clusters (*k*) for each IL-1R1-ECD form, and the clusters were ranked by the number of structures. The silhouette analyses for the soluble and the membrane-bound IL-1R1-ECDs is illustrated in Supplementary Figure S10. In the case of the soluble ECD, the best silhouette width value was obtained for eight clusters, whereas *k* = 4 provided the best score for the membrane-bound counterpart. Importantly, this standard metric provides evidence for a wide dispersal of linker orientations in the soluble system, and the need to choose a higher *k* to attain an optimal partitioning of the sampled dihedral angles given that the flexible loop underlines the highest conformational freedom of this IL-1R1-ECD form.

Among the eight clusters extracted from the soluble ECD simulations (Figure 6A), backbone dihedral angle populations accounting for the closed IL-1R1-ECD conformations are retrieved across five clusters—1S (21.9%), 3S (18.2%) 5S (10.3%), 7S (4.3%) and 8S (2.6%). Clusters 1S, 3S and 8S comprise the closed states prevalent across the S2 and S3 trajectories, whereas clusters 5S and 7S contain the structurally distinct closed conformations adopted by the soluble IL-1R1-ECD throughout the S5 trajectory. The major difference between these two groups is found at the orientation of the Phi (φ) angle of Asn204, which differs significantly in them (Figure 7A). We hypothesize that during the transition from open to closed IL-1R1-ECD, the change in the Asn204φ angle causes the linker in 5S and 7S to twist, allowing D3 to rotate relative to D1-D2, yielding twisted-closed conformations. Indeed, the backbone ϕ angle adopted by Asn204 in the 5S and 7S conformations, respectively characterized by 85° and 110° rotations, agrees well with the Asn204φ angle measured in the closed IL-1R1-ECD X-ray structure (PDB entry 1G0Y), where D3 appears rotated by almost 170 degrees relative to the first two domains of the receptor. The majority of the linker orientations found in IL-1R1-ECD closed conformations expose significant changes to the Glu202ψ and/or Glu203ψ angles (Figure 7B), suggesting that the open-to-closed conformational transition is dependent on changes to these dihedral angles. The remaining clusters, 2S (21.7%), 4S (15.3%) and 6S (5.9%), are densely populated with open-twisted IL1R1-ECD conformations. As evident from Figure 7D, residue Glu203ψ and Asn204ψ angles show relatively large changes in these conformations, when compared to the open IL-1R1-ECD X-ray structure (PDB entry 4GAF). Accordingly, variations in these dihedral angles may drive the conformational alteration to open-twisted states.

For the membrane-bound IL-1R1-ECD, the distribution of linker orientations is different to that of soluble ECD (Figure 6B). The most populated cluster (1M) in the membrane-bound system comprises approximately 62.0% of backbone dihedral angles defining open conformations similar that of the open-state crystallographic structure. Indeed, backbone dihedral angles of 1M largely agree with the experimental data in this regard (Figure 7C,D). The population of dihedral angles representing closed ECD conformations is associated to cluster 2M, containing 16.5% of the total ϕ and ψ angles. This cluster recapitulates similar ϕ and ψ angle distributions, as observed in clusters 1S and 3S (Figure 7A,B). Moreover, from the analyses it can be appreciated that the linker of the membrane-bound IL-1R1-ECD system samples a narrower range of closed conformations, as compared to the soluble form. Clusters 3M and 4M encompass backbone dihedral angles orientations accounting for open-twisted conformations, containing 11.8% and 10.2% of the total population, respectively. A comparable pattern of changes in Glu203ψ and Asn204ψ angles was found in these linker orientations, consistent with the observations made on the soluble clusters 2S, 4S and 6S (Figure 7D).

Overall, the comparison of backbone dihedral angles (φ, ψ) of the five residues composing the flexible linker of the soluble and the membrane-bound IL-1R1-ECD suggests that the former samples a wide array of conformational populations, farther away from its starting point, whereas the membrane-bound IL-1R1-ECD seems to undergo more limited structural deviations. Therefore, it can be reasoned that the conformational space allowed by backbone dihedral angles is more restricted in the membrane-bound IL-1R1-ECD.

## 3. Discussion

The IL-1R1 signaling pathway stands out as one of the major drivers and regulators of the inflammatory status required for host defense and survival against immune challenges. However, persistent activation of the IL-1/IL-1R1 complex is intimately linked with the pathogenesis of a plethora of disease states, such as rheumatoid arthritis, type 2 diabetes, autoinflammatory diseases, cancer and neuroinflammation-associated neurodegenerative diseases. A detailed understanding and characterization of the ensemble of conformations explored by both the soluble and membrane-bound IL-1R1-ECDs may provide novel insights at the molecular level into the structure and dynamics of the receptor, its biology and eventually druggability.

In this contribution, we conducted all-atom MD simulations, totaling 6 μs, of IL-1R1 in a soluble form and a membrane-bound form inserted in a POPC lipid-bilayer to compare the conformational landscape explored by the ectodomain (ECD) of both forms. The comparative conformational analysis of the two IL-1R1-ECD forms suggests that the transition from open to closed states throughout the MD trajectories does not occur in a similar fashion for both protein forms—as imprinted in their Cα-RMSD profiles. PCA analysis reveals that the membrane-bound ECD visits more intermediate open conformations than the soluble ECD, before fully transitioning to the closed state, whereas the open-to-closed transition of the soluble IL-1R1-ECD is less populated by intermediate states. While both soluble and membrane-bound forms are able to sample a large population of open ECD conformations, our simulations demonstrate that the soluble receptor is able to explore to a higher extent the rotational flexibility enabled by the linker connecting D2 and D3. By contrast, membrane-bound IL-1R1-ECD exhibits reduced conformational mobility. We thus contemplate that the distinct conformational pathways observed are most likely induced by different degrees of D3 mobility via the flexible linker. In this sense, membrane anchoring and the close contact between the ECD of IL-1R1 and the lipid-bilayer surface seems to limit the conformational dynamics of the IL-1R1-ECD, when compared to the soluble form.

The flexibility of protein loops, i.e., the ability to adopt multiple conformations, is often critical to biological function and molecular recognition. These flexible, short regions allow significant hinge movements of structural domains, while maintaining the individual domains’ 3D fold. To understand these movements in the IL-1R1-ECD, we measured the deviations in the hinge angles of the two ECD forms. We observed considerable differences in the overall distribution of hinge angles, revealing a wider mobile hinge-like motion of the soluble IL-1R1-ECD. Indeed, the hinge angles between D1-D3 ranged from 110° to 180°, compared to 120° to 172° angles occurring in the membrane-bound ECD. Together with the analysis of structural properties, these results suggest that the soluble and membrane-bound forms could favor the open, closed, and intermediate conformations of the ECD differently, which could reflect on different ligand recognition patterns and, hence, biological activity.

From our PCA analysis, the good overall agreement of our simulations results with experimental data suggests that we have captured much of the essential structural dynamics of the two IL-1R1-ECD forms in the first two principal components (PCs), accounting for more than 85% of the total variation. Still, a notable difference between the soluble and membrane-bound IL-1R1-ECDs stand out when looking to PC3 and PC4, indicating different twisting motion patterns and low structural alignment with the IL-1R1-ECD structures solved experimentally. These differences point to distinct arrangements of the flexible linker as the basis for the markedly different structure-dynamics relationships between the two IL-1R1 forms and the experimental X-ray structure. With this in mind, the linker conformations sampled from the MD simulations were clustered based on the backbone dihedral angles (φ, ψ) of this region. For the soluble IL-1R1-ECD, a higher number of clusters were obtained, reflecting the extensive conformational diversity of the soluble system and suggesting a higher intrinsic interdomain flexibility of the linker in this IL-1R1 form. The dihedral angle changes on the closed and open-twisted conformations were similar in both forms: (i) the major contributions for closed conformations came from deviations on Glu202ψ and/or Glu203ψ angles; and (ii) changes in Glu203ψ and Asn204ψ torsion angles were mostly correlated with the adoption of open-twisted IL-1R1-ECD states. Interestingly, the soluble ECD features linker orientations leading to twisted-closed conformations characterized by 85° and 110° D3 rotations relative to D1-D2. Contributions to these conformations came in a great extent by changes in the Asn204φ angle. Given the resemblance of these conformations with the twisted-closed IL-1R1-ECD crystallographic structure, we may speculate that this conformation approaches a minimum energy state.

The most relevant question of our work is arguably whether, and if so, how, the soluble form differs from the membrane-bound IL-1R1-ECD, in particular in relation to its structural and conformational preferences. It is concluded that major differences between the soluble and membrane-bound IL-1R1-ECDs appear to be mainly governed by conformational rearrangements of the linker connecting the D2 and D3 domains. We hypothesize that, in the soluble environment and in the absence of the structural restraints imposed by the lipid-bilayer, the φ/ψ torsional angles of the D2-D3 linker have a wider range of accessible values, whereas when anchored to a membrane the torsion angles of the linker backbone are limited to a smaller set of possibilities, yielding limited and distinct IL-1R1-ECD conformations. As both forms of IL-1R1 occur physiologically, our results contribute to a better understanding of the distinct structure-dynamics behaviors of the soluble versus membrane-bound forms of IL-1R1, which may be of particular relevance in the context of therapeutic targeting of both IL-1R1-ECD forms.

## 4. Materials and Methods

This section describes the material and methods employed in (1) the preparation and refinement of the soluble IL-1R1-ECD structural model; (2) the prediction and refinement of a 3D-model for the full-length IL-1R1; (3) the attachment of the resultant full-length IL-1R1 model to a POPC (1-palmitoyl-2-oleoyl-sn-glycero-3-phosphocholine) membrane; (4) the setup of all-atom MD simulations; and (5) the analyses of the MD trajectories including the principal component analysis (PCA) and clustering based on the backbone dihedral angles. The sequence of human IL-1R1 (*Homo Sapiens*, UniProt code P14778) was used to create the full-length IL-1R1 protein by means of structural modeling methodologies. The quality of the structural models was evaluated using PROCHECK v.3.5.4 [16]. MD simulations were performed on a HPC cluster (600 computing nodes comprising dual Intel^®^ Xeon^®^ CPU E5-2680 @2.70GHz), using message passing interface (MPI) parallelization and managed by Minho Advanced Computing Centre (MACC, Braga, Portugal). Performance/run on 160 cores (16 cores/node) was around 54ns/day (soluble system) and 50ns/day (membrane-bound system). MD simulation data were visualized using VMD v.1.9.3. [24] and analyzed with RStudio 1.2.5033 [25]. The BlendMol plugin [26] was used to produce high quality images of IL-1R1 structures from imported VMD visualization states using the Blender software [27], an open-source 3D-modeling and rendering program.

### 4.1. Protein Structure of Soluble IL-1R1 (ECD)

As of December 2021, the PDB [28] holds 5 crystal structures of protein complexes containing the IL-1R1-ECD, with 100% coverage of the UniProt sequence encoding for the human IL-1R1-ECD. PDB entry 4GAF (chain B—IL-1R1-ECD) was selected because of its higher structural resolution (2.15 Å) [29]. Then, the co-crystallized ligands and water molecules were removed, missing residues were modeled via structural modeling using MODELLER v9.19 [30], and the best model was selected on the basis of the lowest value of the DOPE score [31]. The final model for soluble IL-1R1 comprises 319 residues.

### 4.2. Structural Assembly and Refinement of a Full-Length IL-1R1

A structural model for the full-length multi-domain IL-1R1 was assembled by combining molecular modeling and MD simulations. The crystal structure of the IL-1R1-bound ECD was obtained from the PDB (chain B from PDB entry 4GAF), determined at 2.15 Å resolution, and prepared as described in 4.1. Next, the 20-residue TM peptide was modeled as a single α-helix via use of a MODELLER script [32], and the PDB entry 1T3G for TIR domain [33] was chosen as template structure for homology modeling by using PDB’s BLAST utility [34]. This crystal structure of the TIR domain of IL-1RAPL1 (Interleukin 1 Receptor Accessory Protein Like 1) holds 34% sequence identity (E-value of 1e-13) with the TIR amino acid sequence of IL-1R1, and has been solved at 2.3 Å resolution [33]. Despite the low sequence similarity, the two proteins belong to the same family of structurally similar receptors, the IL-1 Receptor Family [2], thus both protein domains adopt a similar global fold, despite some differences in the positioning of TIR domain secondary structural elements [35]. For each model, a 1000-step steepest descent energy minimization was carried out to remove initial steric clashes using GROMACS software version 2019.3 [36,37]. All three individual models were aligned on the same axis using GROMACS tool *gmx editconf* and patched between the C- and N-terminal residues of adjacent domains using UCSF Chimera version 1.12 [38]. Finally, the resulting full-length model was again energy minimized with an additional loop refinement step, using the MODELLER software in Chimera [38], to correct irregularities on the constructed peptide bonds. The final full-length IL-1R1 model presents 527 residues (IL-1R1-ECD—319 residues; TM—20 residues; TIR—184 residues).

### 4.3. Construction of the Full-Length IL-1R1 Membrane System

The full-length IL-1R1 model was embedded in a pre-equilibrated POPC bilayer containing 332 lipids by aligning the center of mass of the IL-1R1 TM domain with that of the membrane, using the *gmx mdrun membed* tool in GROMACS 2019.3 [39]. The overlapping lipids with the protein were removed, resulting in a total of 324 membrane lipids. Periodic boundary conditions were applied in all directions of a cubic simulation box of 10 × 10 × 20 nm, the system solvated using the TIP3P water model [40] and neutralized with the addition of 133 Na^+^ and 132 Cl^−^ ions at a 100 mM concentration, leaving a total of 61.930 water molecules. The resulting total number of atoms was 212.017.

### 4.4. MD Simulations of Full-Length IL-1R1 Membrane System

All-atom simulations were performed with GROMACS 2019.3, using the Amber99sb-ILDN force field [41] for the protein and Berger parameters [42] for the POPC lipids. Energy minimization was carried out using the steepest descent algorithm for 50,000 steps to remove close contacts between the IL-1R1 atoms and solvent or lipid bilayer. Equilibration of the protein-membrane system started with a 100 ps simulation under NVT conditions at 303 K. All bond lengths were constrained with the LINCS algorithm [43], with a 2 fs time step. Electrostatic interactions were treated by using the particle-mesh Ewald (PME) method [44], with a short-range cutoff of 12 Å. The cutoff distance of the van der Waals interaction was also of 12 Å. The temperature was coupled to the V-rescale thermostat [45] with a time constant of 0.1 ps. Position restraints were applied first to all heavy atoms, and then backbone, and finally Cα-atoms, using a force constant of 1000 kJ/mol/nm^2^. After NVT, a 10 ns NPT equilibration was performed, with pressure kept constant at 1 bar by using a semi-isotropic Parrinello-Rahman barostat [46]. The temperature was kept at 303 K using the Nose-Hoover thermostat [47], maintaining the position restraints from the NVT phase. After these equilibration steps, restraints were removed and five independent production runs, each for 600 ns, were performed starting from different initial velocities.

### 4.5. MD Simulations of Soluble IL-1R1 (ECD)

MD simulations in explicit solvent were performed for the isolated IL-1R1-ECD, using Amber99sb-ILDN force field [41]. The model was solvated with TIP3P water [40] in a truncated octahedron box of 12 × 12 × 12 nm. The system was neutralized with the addition of 101 Na+ and 98 Cl- ions at a 100 mM concentration. The resulting total number of atoms was 164.708. Next, 10,000 steps of energy minimization were performed with the steepest descent algorithm. Temperature and pressure couplings were performed for 750 ps and 1 ns each, with V-rescale thermostat [45] and Berendsen barostat [48], respectively. All other MD simulation settings were the same as described for the full-length IL-1R1 membrane system. Production simulations of 600 ns were performed in quintuplicate, starting from different initial velocities.

### 4.6. Principal Component Analysis

PCA was performed to extract the collective motions of the IL-1R1-ECD sampled in the MD simulations. The covariance matrix was constructed using the *gmx covar* function in GROMACS 2019.3 to define the backbone conformational space of the MD structures. Next, the MD trajectories were projected onto the first four eigenvectors, using 10,000 frames per trajectory (1 frame each 60 ps), with an initial RMS-fit to the starting MD snapshot Cα-atoms to remove the rotational and translational motions. To understand the global motions of the IL1R1-ECD, porcupine plots were generated and visualized via the modevector.py script from Pymol [49]. Five crystal structures of IL-1R1-ECD bound to different ligands were used in the PCA as reference points in the conformational space sampled by the two IL-1R1-ECD forms—PDB entries 4DEP two ECD chains [8], 4GAF [29], 1G0Y [11], 1IRA [50] and 1ITB [10].

### 4.7. Clustering Based on the Backbone Dihedral Angles of the Flexible Linker

Conformational clustering based on the backbone dihedral angles, phi (φ) and psi (ψ), of the flexible linker residues was performed using the partitioning around medoids (PAM) clustering method [51]. Proline (Pro) 206 was not included due to limited number of ψ and φ possibilities owed to its 5-membered ring. The silhouette width value was used to select the best number of clusters obtained with the PAM algorithm. This value measures the quality of a clustering, where the optimal number of clusters *k* is the one that maximizes the average silhouette over a range of possible values for *k* [52]. Representative structures of each cluster were chosen based on the medoids concept, i.e., structures closest to the absolute average of each cluster. Clustering was performed using 10,000 frames of the linker region per MD trajectory (1 frame each 60 ps), after alignment of the Cα-atoms in each frame to the starting MD snapshot.

## 5. Conclusions

The interleukin-1 receptor type 1 (IL-1R1) is a well-established player in immune and inflammation pathways, and unraveling its structural and dynamics complexities could shed light on the biology of this receptor. The results presented herein provide a comprehensive picture of the dynamics of the flexible D2/D3 linker present on the soluble and membrane-bound forms of the IL-1R1 ectodomains (ECDs), as well as the range of spatial orientations and relationships the Ig-like domains of the ECD may populate. The soluble form of this cytokine receptor is a rather dynamic and flexible protein whose activity has been linked structurally to the interdomain flexibility of the D2/D3 linker. In the presence of a biological membrane, the flexibility of the linker region appears more restrained, limiting the conformational freedom of the Ig-like domains and therefore may play a differential role in the mechanisms of cytokine recruitment and signaling. These results may be further exploited to study the druggability of each of the IL-1R1 forms and thus develop novel strategies for the therapeutic modulation of IL-1 signaling.

## Figures and Tables

**Table 1:** Average values of multiple structural properties across five MD trajectories of soluble IL-1R1-ECD (S1, S2, S3, S4 and S5) and five MD trajectories of POPC-anchored IL-1R1-ECD (M1, M2, M3, M4 and M5). Reference values computed for the open (PDB entry 4GAF) and closed (PDB entry 1G0Y) crystal structures are presented.

System	Average RMSD (Å)	R_g_ (nm)	# HB_intra_	SASA (nm^2^)
4GAF ^1^	--	3.0	229	179.4
1G0Y	16.8	2.2	249	174.4
S1	18.8 ± 0.22	2.9 ± 0.17	234 ± 7	178.7 ± 4.25
S2	16.5 ± 0.02	2.2 ± 0.06	240 ± 8	173.0 ± 2.74
S3	16.9 ± 0.04	2.2 ± 0.04	241 ± 7	172.7 ± 3.13
S4	20.6 ± 0.24	2.8 ± 0.20	233 ± 7	178.9 ± 3.77
S5	15.6 ± 0.20	2.4 ± 0.19	234 ± 8	176.4 ± 4.50
M1	14.1 ± 0.07	2.3 ± 0.18	239 ± 7	175.8 ± 4.28
M2	17.3 ± 0.13	3.1 ± 0.08	231 ± 7	184.7 ± 2.88
M3	11.7 ± 0.11	3.3 ± 0.15	232 ± 7	186.3 ± 2.60
M4	15.6 ±0.18	3.0 ± 0.15	233 ± 8	183.2 ± 3.13
M5	14.1 ±0.06	2.3 ± 0.18	238 ± 7	175.3 ± 4.35

^1^ reference structure used as starting point for MD simulations. Table abbreviations: root mean square deviation (RMSD); radius of gyration (Rg); intramolecular hydrogen bonds (HB_intra_); solvent-accessible surface area (SASA).

## Data Availability

The data presented in this study are available on request from the corresponding author.

## Supplementary Materials

The following is available online at https://www.mdpi.com/article/10.3390/ijms23052599/s1.
