# Supplementary material for: Conformational Dynamics of the Soluble and Membrane-Bound Forms of Interleukin-1 Receptor Type-1: Insights into Linker Flexibility and Domain Orientation
Source: Int J Mol Sci. 2022 Feb 26;23(5):2599. doi: 10.3390/ijms23052599 (PMC8910350; doi:10.3390/ijms23052599)
Supplement: Supplementary file 1 [file ijms-23-02599-s001.zip › ijms-1547387-supplementary.pdf]

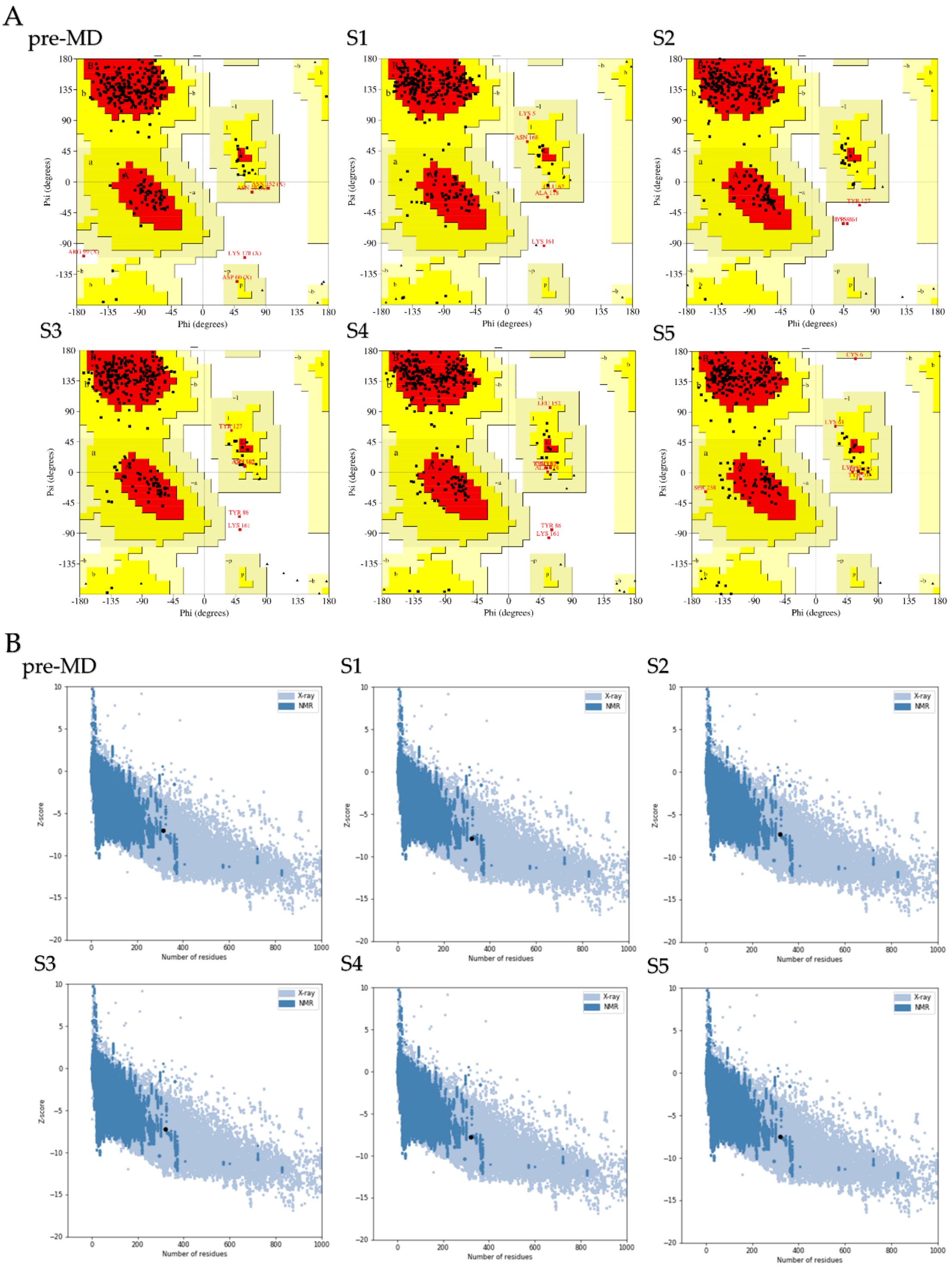

Figure S1. (A) Ramachandran plot for the pre-MD model and the final snapshots of the S1, S2, S3, S4 and S5 soluble IL-1R1 trajectories, demonstrating that the protein backbone angles remain within the favoured regions of the Ramachandran plot (B) *Z*-plot for the pre-MD model and the final snapshots of the S1, S2, S3, S4 and S5 soluble IL-1R1 trajectories. Z-scores for all models (represented in the black dots) were found to be within the range typically observed for proteins determined by X-ray crystallography (light blue) and NMR spectroscopy (dark blue).

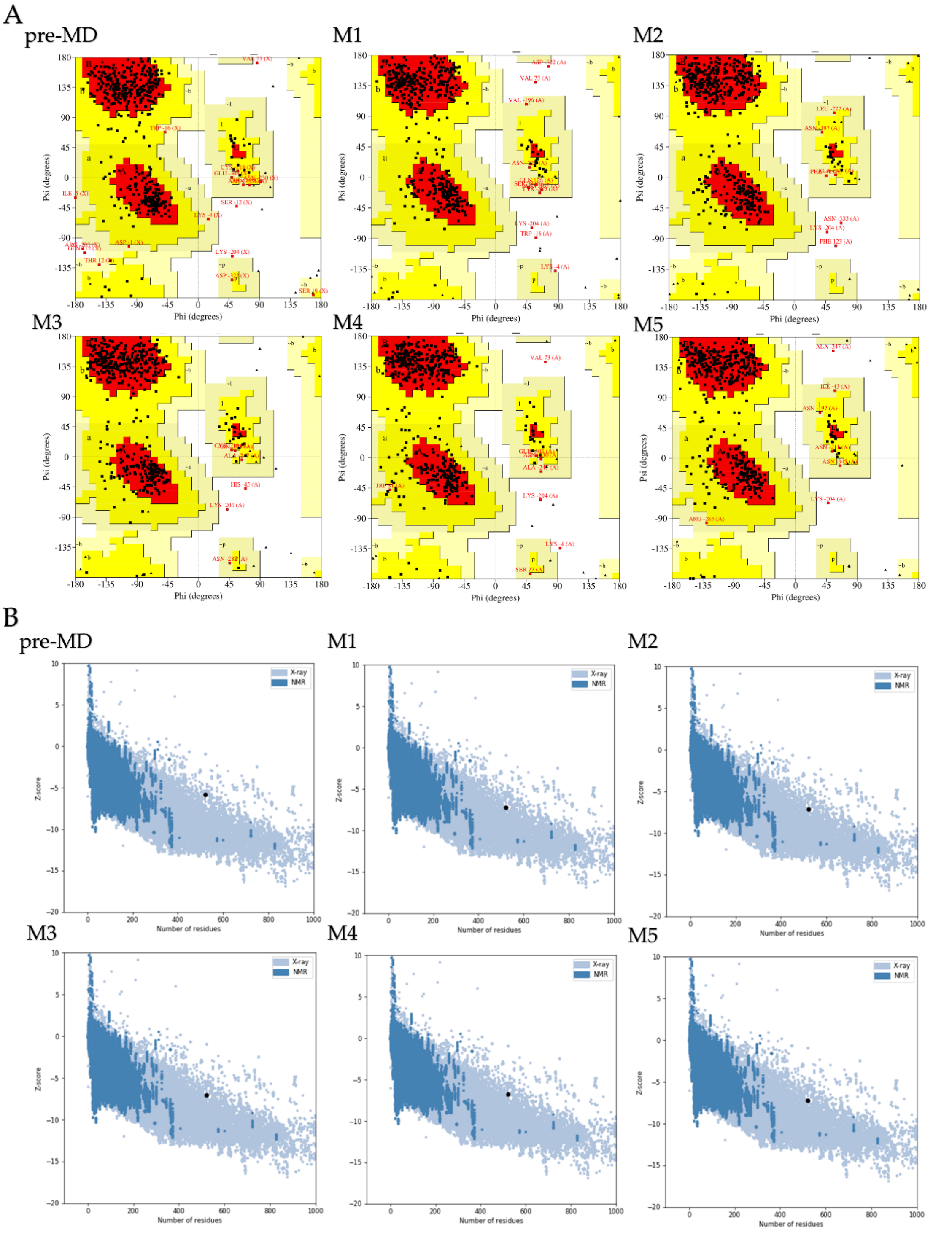

Figure S2. (A) Ramachandran plot for the pre-MD model and the final snapshots of the M1, M2, M3, M4 and M5 membrane-bound full-length IL-1R1 trajectories, demonstrating that the protein backbone angles remain within the favoured regions of the Ramachandran plot (B) Z-plot for the pre-MD model and the final snapshots of the M1, M2, M3, M4 and M5 membrane-bound full-length IL-1R1 trajectories. Z-scores for all models (represented in the black dots) were found to be within the range typically observed for proteins determined by X-ray crystallography (light blue) and NMR spectroscopy (dark blue).

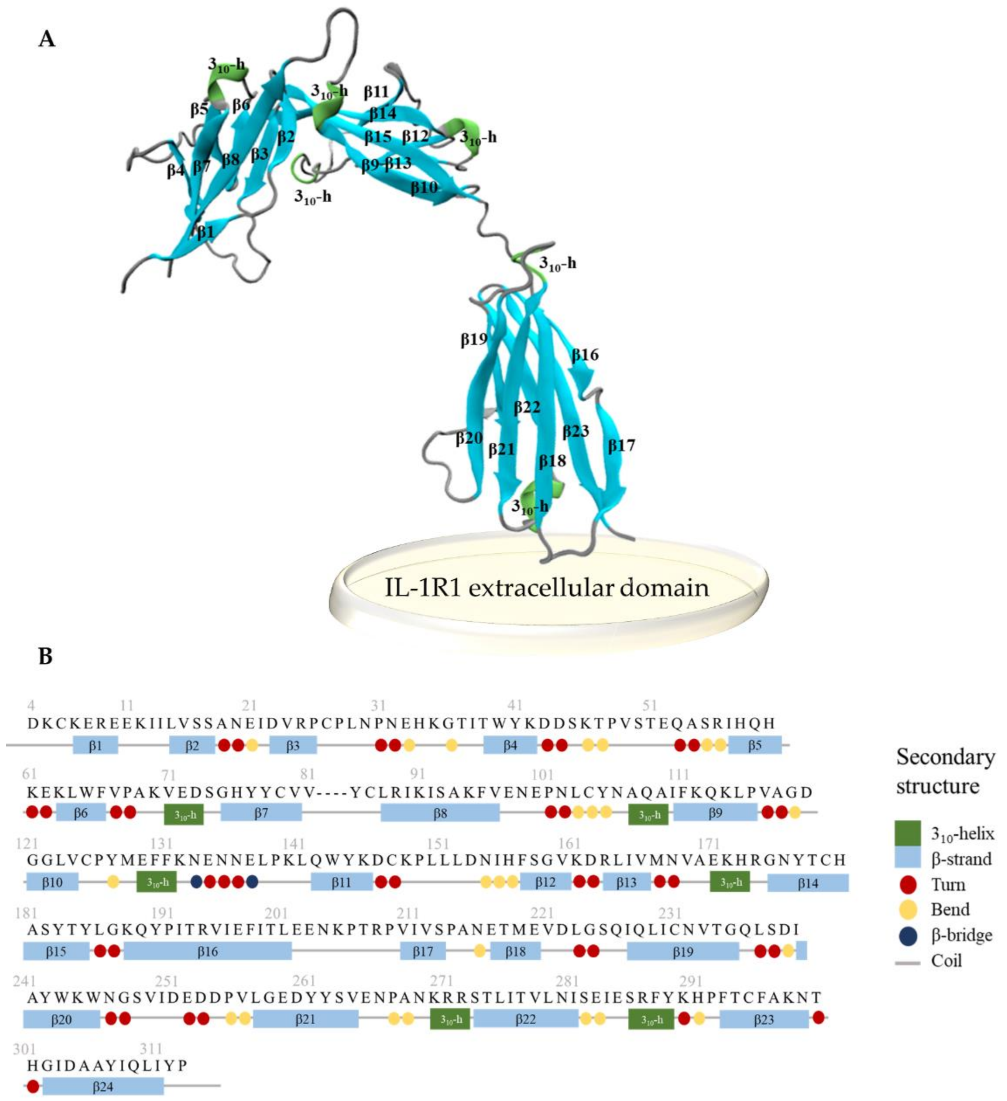

Figure S3. Secondary structure assignment of the extracellular domain of interleukin-1 receptor type 1 (IL-1R1-ECD, PBD entry 4GAF). (A) Cartoon representation of the crystallographic structure of the IL-1R1-ECD and (B) position of secondary structure elements across the IL-1R1-ECD sequence. The secondary structure was assigned using the DSSP algorithm. The secondary structural elements are labelled according to their occurrence in sequence: β-strands (coloured in blue), 3_{10}-helices (coloured in green), turns (coloured in red), bends (coloured in yellow), β-bridge (coloured in dark blue) and coils (coloured in gray).

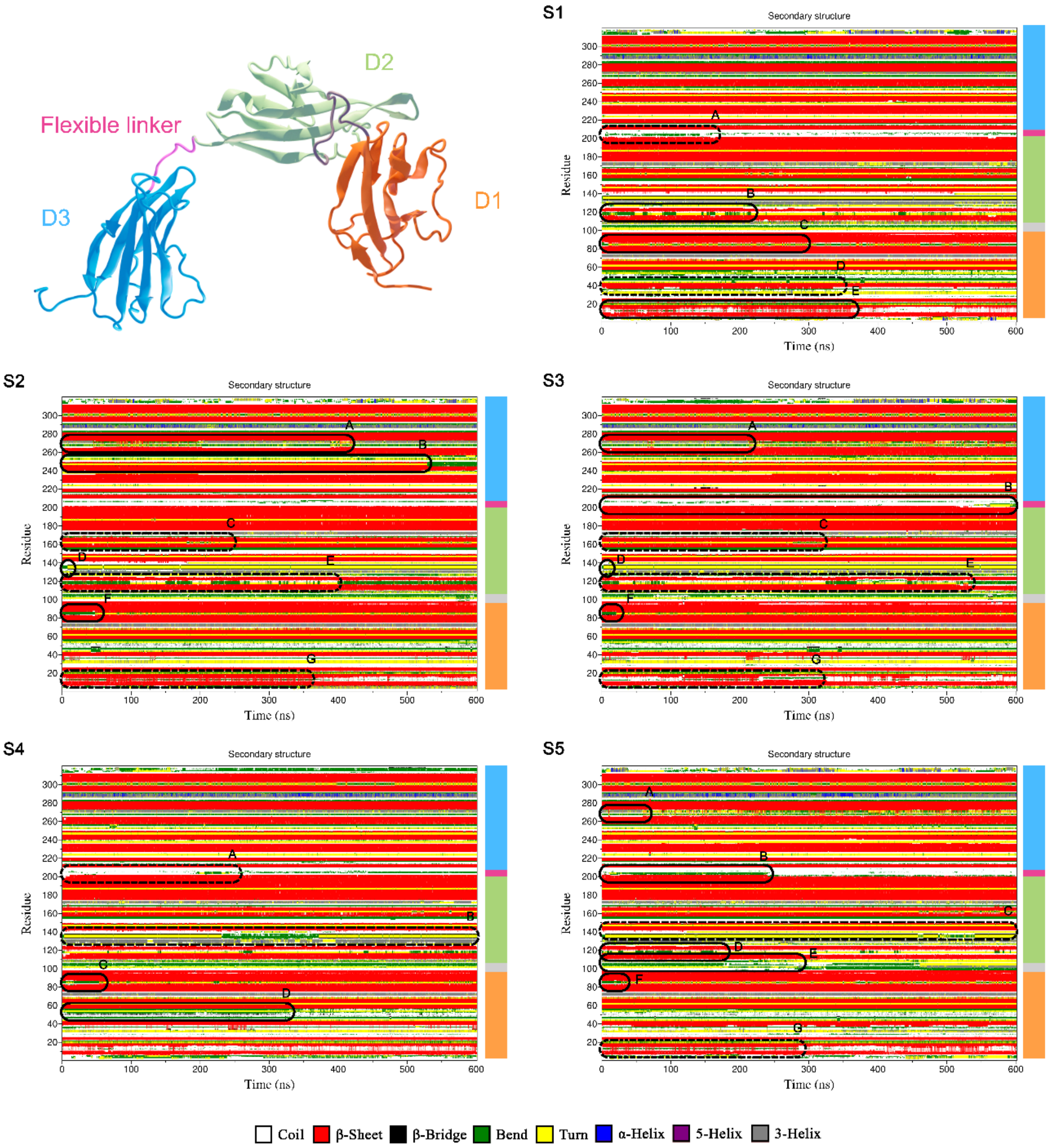

Figure S4. Secondary structure assignment according to DSSP, throughout the whole simulation length for the soluble IL-1R1-ECD (S1, S2, S3, S4, and S5), revealing that the three Ig-like domains are mostly preserved throughout the simulation timescale. Significant variations are circled in black: solid lines represent relevant changes in the secondary structure; dashed lines indicate smaller variations that tend to reset to the original secondary structure.

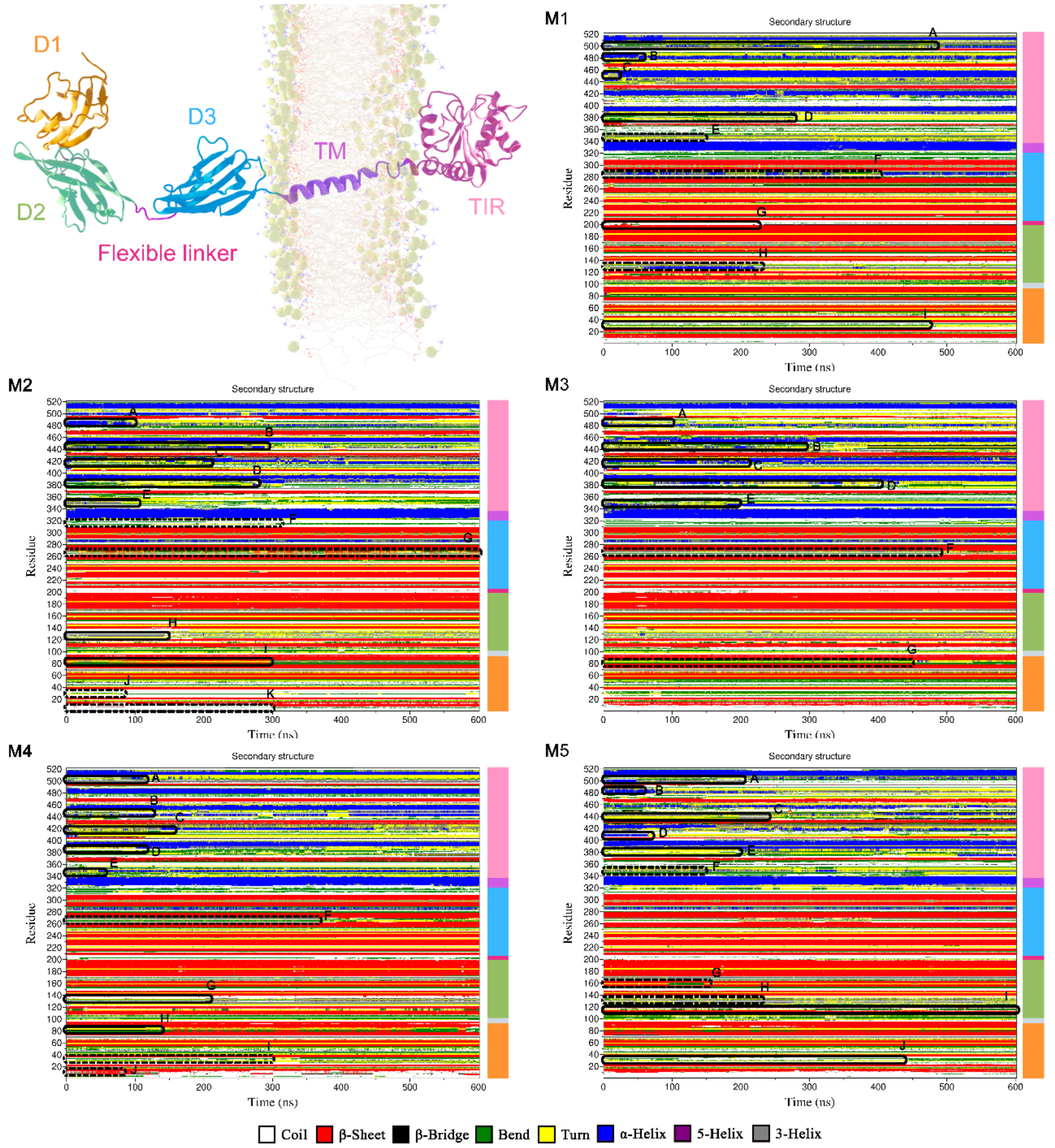

Figure S5. Secondary structure assignment according to DSSP, throughout the whole simulation length for the membrane-bound IL-1R1-ECD, TM and TIR domains (M1, M2, M3, M4, and M5), revealing that the IL-1R1 structure is mostly preserved throughout the simulation timescale. Significant variations are circled in black: solid lines represent relevant changes in the secondary structure; dashed lines indicate smaller variations that tend to reset to the original secondary structure.

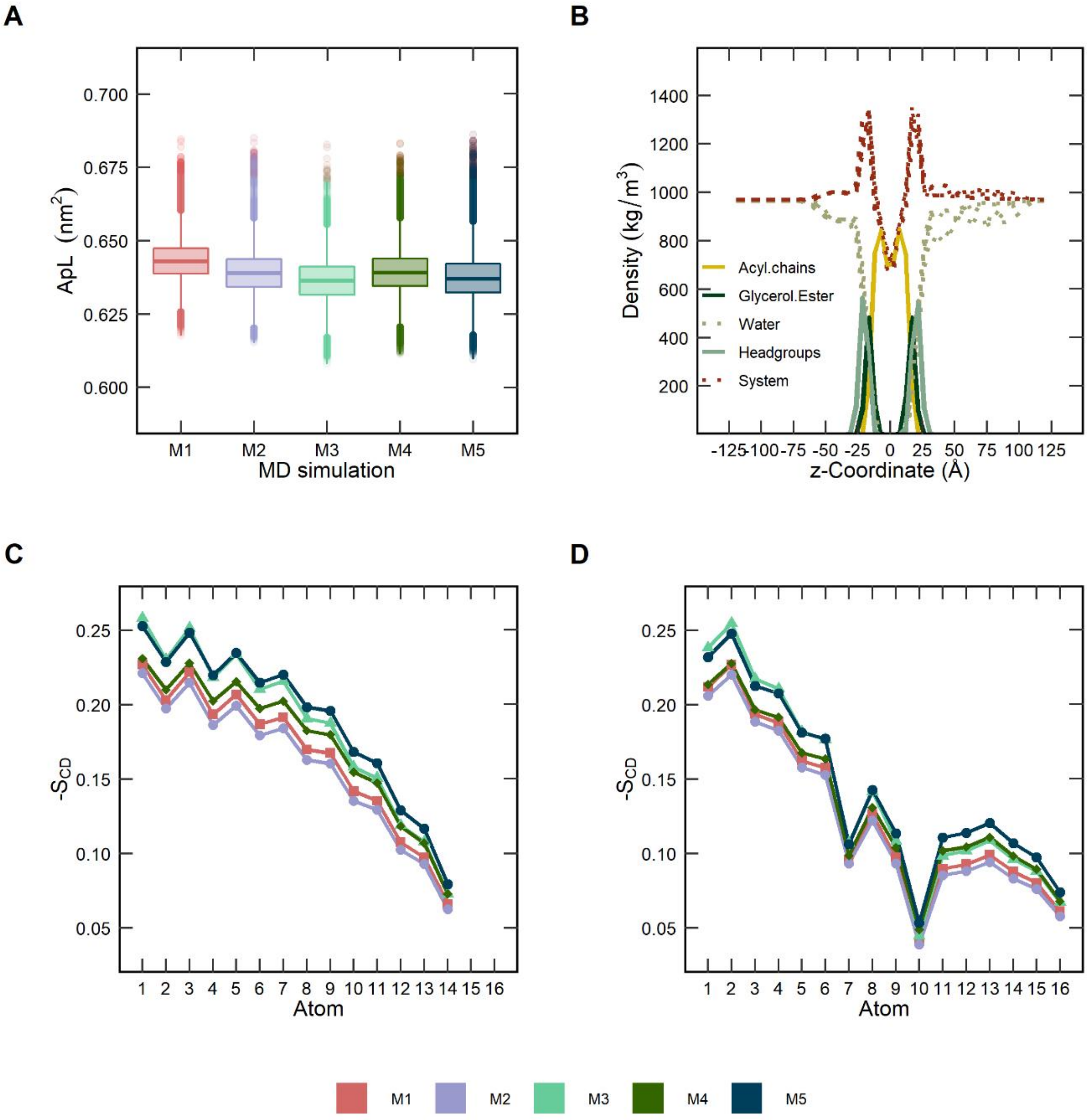

Figure S6. Bilayer stability parameters throughout five MD runs (M1, M2, M3, M4 and M5) of the full-length IL-1R1 in a POPC membrane. (A) Area per lipid (ApL) of the POPC bilayer; (B) Mass density profiles of the various components of the POPC bilayer; Deuterium order parameter S_{CD} profiles of the *sn1* (C) and *sn2* (D) lipid acyl chains;

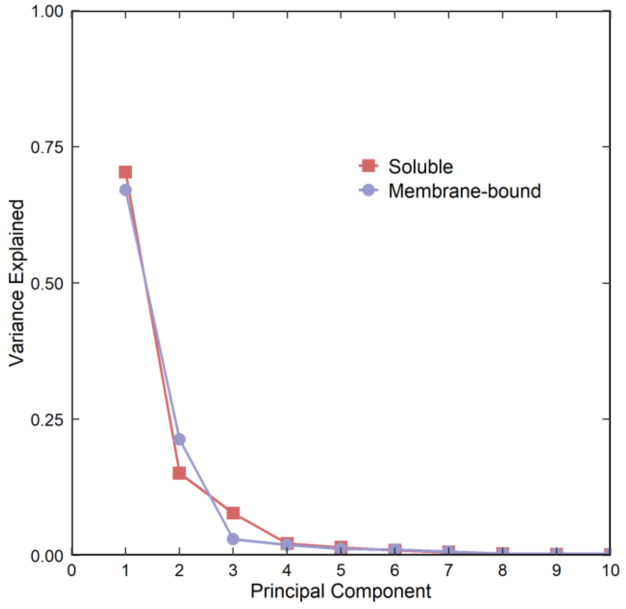

Figure S7. Scree plot representing the proportion of variance explained for the first 10 principal components, determined by the PCA of the (A) soluble (red line) and (B) membrane-bound (purple line) IL-1R1-ECDs MD simulations.

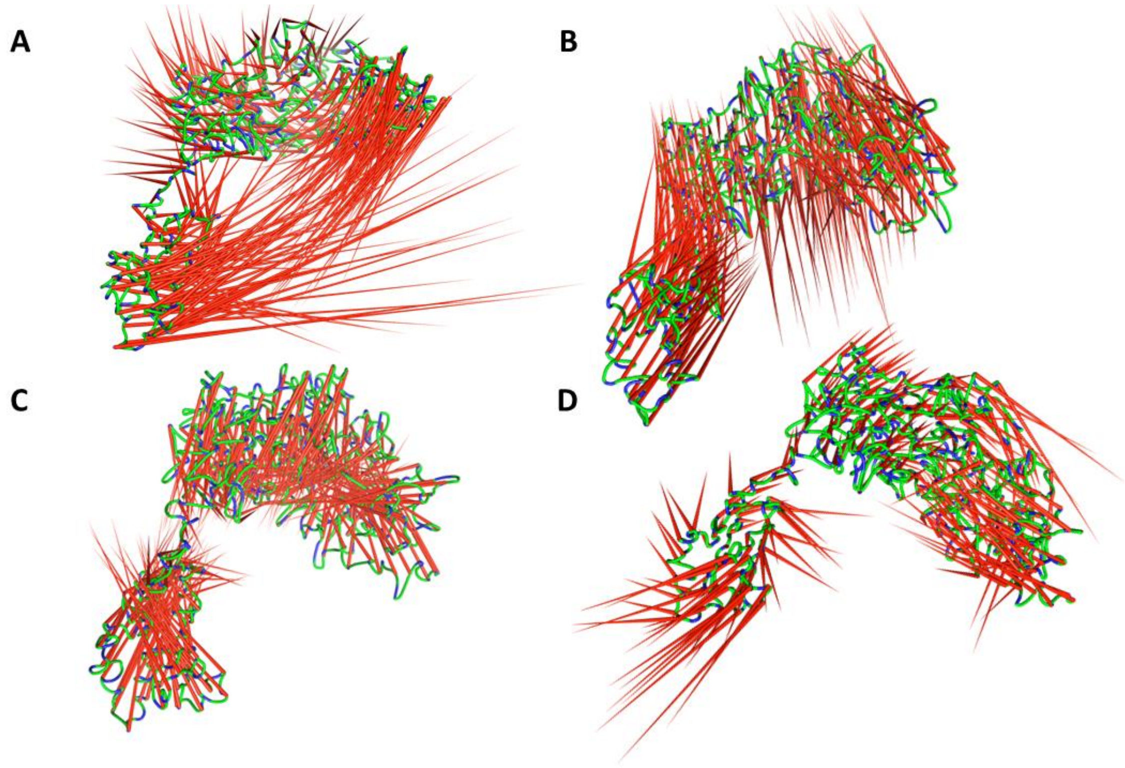

Figure S8. Porcupine plots showing the significant motion across (A) PC1; (B) PC2; (C) PC3; and (D) PC4, in the IL-1R1-ECD. The arrows indicate the direction of correlated motion.

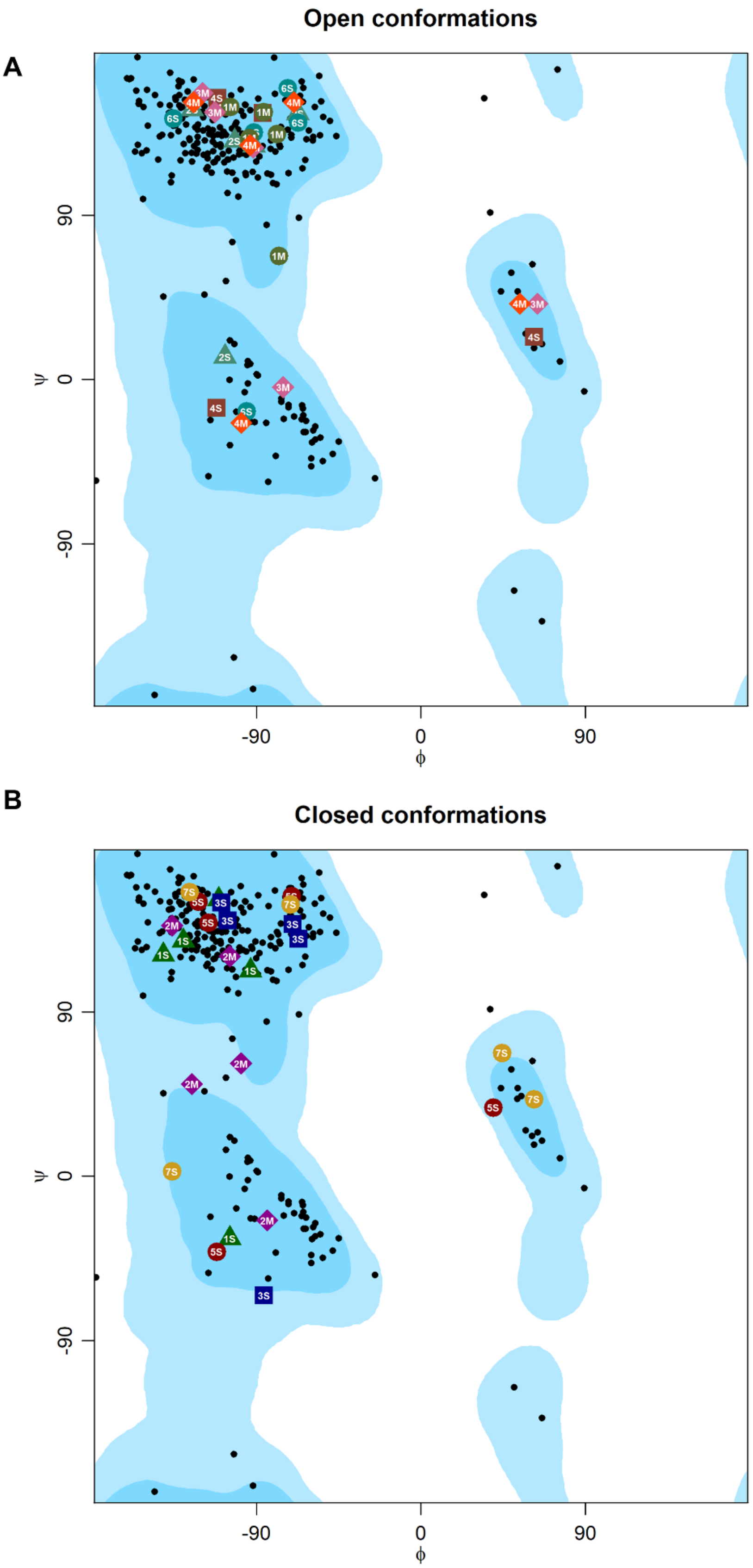

Figure S9. Superposition of the linker backbone dihedral angles, ϕ and ψ, for the (A) open and (B) closed representative conformations of the extracellular domain of interleukin-1 receptor type 1 (IL-1R1-ECD), onto the Ramachandran plot of the crystallographic structure of IL-1R1-ECD (PDB entry 4GAF).

Represented clusters of soluble ECD: Cluster 1 [1S] (dark green); Cluster 2 [2S] (light green); Cluster 3 [3S] (dark blue); Cluster 4 [4S] (brown); Cluster 5 [5S] (dark red); Cluster 6 [6S] (cyan). Represented clusters of membrane-bound ECD: Cluster 1 [1M] (dark green); Cluster 2 [2M] (purple); Cluster 3 [3M] (mauve); Cluster 4 [4M] (orange).

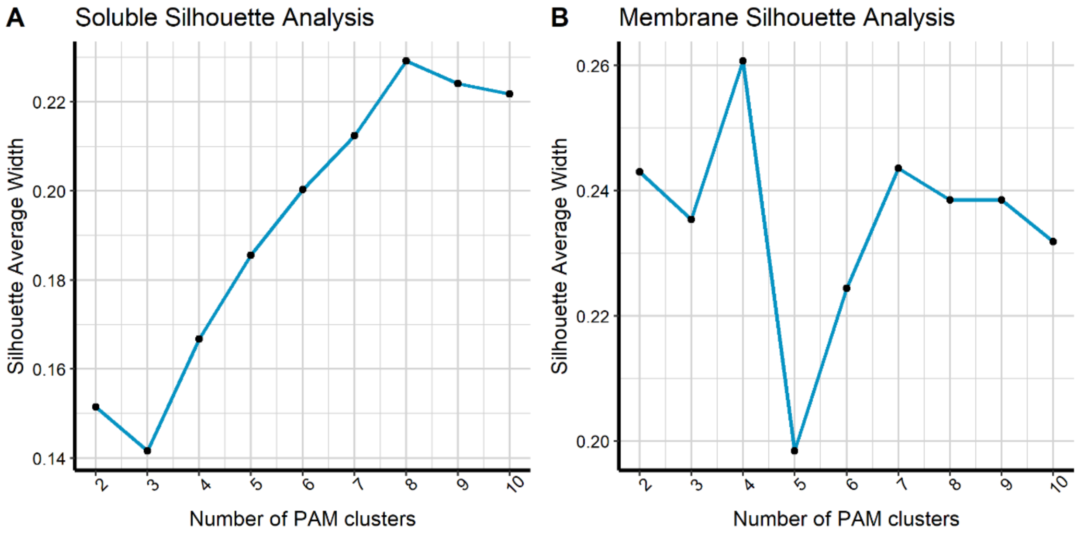

Figure S10. Average silhouette width of different number of clusters (*k*) for the backbone dihedral angles (φ, ψ) of the flexible linker connecting D2 and D3 domains. Silhouette scores for (A) the soluble and (B) the membrane-bound IL-1R1-ECD.

Table S1. Ramachandran plot statistics and ProSA-web Z-score for initial structural models and the five MD runs of soluble and membrane-bound full-length IL-1R1.

IL-1R1 protein		Ramachandran plot[1]				ProSA-web Z-score
		Most favoured regions	Additional allowed regions	Generously allowed regions (%)	Disallowed regions (%)	
**Soluble**	Pre-MD	250 (88.7%)	27 (9.6%)	3 (1.1%)	2 (0.7%)	-7.06
	S1[2]	243 (85.0%)	38 (13.3%)	4 (1.4%)	1 (0.3%)	-7.87
	S2[2]	253 (88.5%)	30 (10.5%)	0 (0%)	3 (1.0%)	-7.34
	S3[2]	239 (83.6%)	42 (14.7%)	3 (1.0%)	2 (0.7%)	-7.24
	S4[2]	238 (83.2%)	41 (14.3%)	5 (1.7%)	2 (0.7%)	-7.75
	S5[2]	237 (82.9%)	43 (15.0%)	5 (1.7%)	1 (0.3%)	-7.53
**Full-length**	Pre-MD	385 (82.4%)	65 (13.9%)	12 (2.6%)	5 (1.1%)	-5.81
	M1[2]	396 (84.8%)	60 (12.8%)	6 (1.3%)	5 (1.1%)	-7.25
	M2[2]	388 (83.1%)	72 (15.4%)	4 (0.9%)	3 (0.6%)	-7.16
	M3[2]	392 (83.9%)	69 (14.8%)	4 (0.9%)	2 (0.4%)	-6.99
	M4[2]	386 (82.7%)	73 (15.6%)	5 (1.1%)	3 (0.6%)	-6.73
	M5[2]	388 (83.1%)	72 (15.4%)	5 (1.1%)	2 (0.4%)	-7.19

[1]*Distribution of non-glycine and non-proline residues in the Ramachandran plot*
[2]*Final snapshot from the simulation*
%Total number of non-glycine and non-proline residues in the protein
